# Correlations between heart sound components and hemodynamic variables

**DOI:** 10.1038/s41598-024-59362-3

**Published:** 2024-04-13

**Authors:** Yong-Seok Park, Hyun-Seok Kim, Seung-Ah Lee, Gyu-Sam Hwang, Woosuk Jung, Baehun Moon, Kyu-Min Kang, Woo-Young Seo, Jun-Gol Song, Sung-Hoon Kim

**Affiliations:** 1 Department of Anesthesiology and Pain Medicine, Asan Medical Center, Brain Korea 21 Project, University of Ulsan College of Medicine, 88, Olympic-ro 43-gil, Songpa-gu, Seoul, 05505 Korea; 2 Biomedical Engineering Research Center, Asan Institute for Life Science, Asan Medical Center, Seoul, Korea; 3 Department of Cardiology, Asan Medical Center, University of Ulsan College of Medicine, Seoul, Korea; 4 Department of Anesthesiology and Pain Medicine, Chungnam National University School of Medicine, Jung-gu, Daejeon, Korea; 5 Medical AI Research Team, Signal House Co., Ltd, Seoul, Korea

**Keywords:** Translational research, Cardiology

## Abstract

Although the esophageal stethoscope is used for continuous auscultation during general anesthesia, few studies have investigated phonocardiographic data as a continuous hemodynamic index. In this study, we aimed to induce hemodynamic variations and clarify the relationship between the heart sounds and hemodynamic variables through an experimental animal study. Changes in the cardiac contractility and vascular resistance were induced in anesthetized pigs by administering dobutamine, esmolol, phenylephrine, and nicardipine. In addition, a decrease in cardiac output was induced by restricting the venous return by clamping the inferior vena cava (IVC). The relationship between the hemodynamic changes and changes in the heart sound indices was analyzed. Experimental data from eight pigs were analyzed. The mean values of the correlation coefficients of changes in S1 amplitude (ΔS1amp) with systolic blood pressure (ΔSBP), pulse pressure (ΔPP), and ΔdP/dt during dobutamine administration were 0.94, 0.96, and 0.96, respectively. The mean values of the correlation coefficients of ΔS1amp with ΔSBP, ΔPP, and ΔdP/dt during esmolol administration were 0.80, 0.82, and 0.86, respectively. The hemodynamic changes caused by the administration of phenylephrine and nicardipine did not correlate significantly with changes in the heart rate. The S1 amplitude of the heart sound was significantly correlated with the hemodynamic changes caused by the changes in cardiac contractility but not with the variations in the vascular resistance. Heart sounds can potentially provide a non-invasive monitoring method to differentiate the cause of hemodynamic variations.

## Introduction

Phonocardiography involves the recording of heart sounds. It provides effective information regarding the functioning of heart valves and hemodynamics of the heart^1^. It can potentially detect various heart diseases and monitor the cardiac function^2,3^. Overlooking the long-standing use of esophageal stethoscopes (ESS) for continuous auscultation during general anesthesia, few studies have investigated the use of phonocardiographic data as an indicator of the cardiovascular function or continuous hemodynamic measurements^4–6^. In previous studies, we proposed that phonocardiogram monitoring during surgery could provide effective clinical information as a non-invasive measure of the hemodynamic status^7–9^. In the two studies, we analyzed the time-domain and frequency-domain characteristics of heart sound signals obtained from digitalized recordings of ESS in patients under general anesthesia. The two studies demonstrated that 1) heart sounds varied differently depending on the mechanism of hemodynamic variations, and 2) the variation trend of the heart sound interval index was similar to that of the pulse pressure (PP)^8^.

However, our previous studies were retrospective investigations of patients undergoing surgery. Therefore, it was infeasible to induce hemodynamic variations in a controlled environment owing to the study design and ethical concerns. In addition, the interference of the electrical or acoustic noise due to surgical situations interfered with obtaining a clean signal. To overcome these limitations, we planned to conduct animal experiments using pigs. In this study, we induced hemodynamic variations in the study animals by administering various drugs that affect the myocardial contractility or vascular resistance and determined whether there were differences in the heart sound variations depending on the mechanism of the drugs. In addition, we simulated substantial intraoperative blood loss by restricting the venous return through surgical intervention and measured the variations in the heart sounds during the process. The purpose of these experiments and analyses was to quantitatively determine the relationship between the heart sounds and hemodynamic variables and identify the differences in heart sound variations caused by different mechanisms of hemodynamic variations.

## Methods

### Study design, subjects, and experiment preparation

This experiment was reviewed and approved by the Institutional Animal Care and Use Committee of the Asan Medical Center (Protocol No. 2020-12-114) and is reported in accordance with ARRIVE guidelines. All methods were performed in accordance with the relevant guidelines and regulations including Laboratory Animal Act and Animal Protection Act. The study was designed as an experimental animal study to analyze the relationship between heart sounds and hemodynamic changes. Since the Pearson correlation is the primary method of analysis, the required sample size was calculated as follows: A type I error probability of α = 0.05 and a type II error probability of β = 0.2 were used. The expected correlation coefficient was assumed to be r = 0.8^10^. Accordingly, Z_α_ (the standard normal deviation for α) = 1.960 and Z_β_ (the standard normal deviation for β) = 0.842. C = 0.5 * ln[(1 + r)/(1−r)] = 1.099, so the required sample size is N = [(Z_α_ + Z_β_)/C]2 + 3 = 10. As a result, ten male Yorkshire pigs were selected as the experimental subjects (weight, 35.5 ± 0.8 kg; age, 3–4 month; source, Kukje Experimental Animal Center, Pocheon-si, Gyeonggi-do, Republic of Korea). As this study analyzes changes in hemodynamic parameters over time, there was no control group. Anesthesia was induced by intramuscular injection of Zoletil™ (zolazepam + tiletamine) 5 mg/kg and xylazine 2 mg/kg. After endotracheal intubation, mechanical ventilation was applied, and anesthesia was maintained by inhalation of 1%vol isoflurane. An intravenous catheter was placed for fluid and drug administration. An arterial catheter was inserted into the femoral artery for arterial blood pressure (ABP) monitoring. After the ESS was inserted into the esophagus, an X-ray was captured to adjust the depth so that the tip of the ESS was positioned at the center of the heart shadow.

### Interventions to induce hemodynamic changes

After anesthesia and the preparation for the experiment were complete, dobutamine was administered with continuous intravenous (IV) infusion at a rate of 10 mcg/kg/min for 10 min to induce an increase in myocardial contractility. A 20% or greater increase in heart rate from baseline was considered the threshold for efficacy, and the dose was doubled when this was not met. In the next phase, esmolol 0.5 mg/kg single IV dose induced a decrease in contractility. The threshold for efficacy was a 20% or greater reduction in heart rate from baseline, and if this was not met, the drug was re-administered at a double dose. Phenylephrine 1 mcg/kg IV was administered to induce an increase in vascular resistance in the third phase. Then, nicardipine 10 mcg/kg IV induced a decrease in vascular resistance in the fourth phase. In the final phase, the inferior vena cava (IVC) was clamped to limit the venous return to simulate a decrease in the cardiac output owing to an acute blood loss. An interval of at least 20 min was permitted between each phase to minimize the effects of the previous -phase's administration and return the hemodynamic status to the baseline.

### Signal acquisition, processing, and analysis

**Figure 1:**
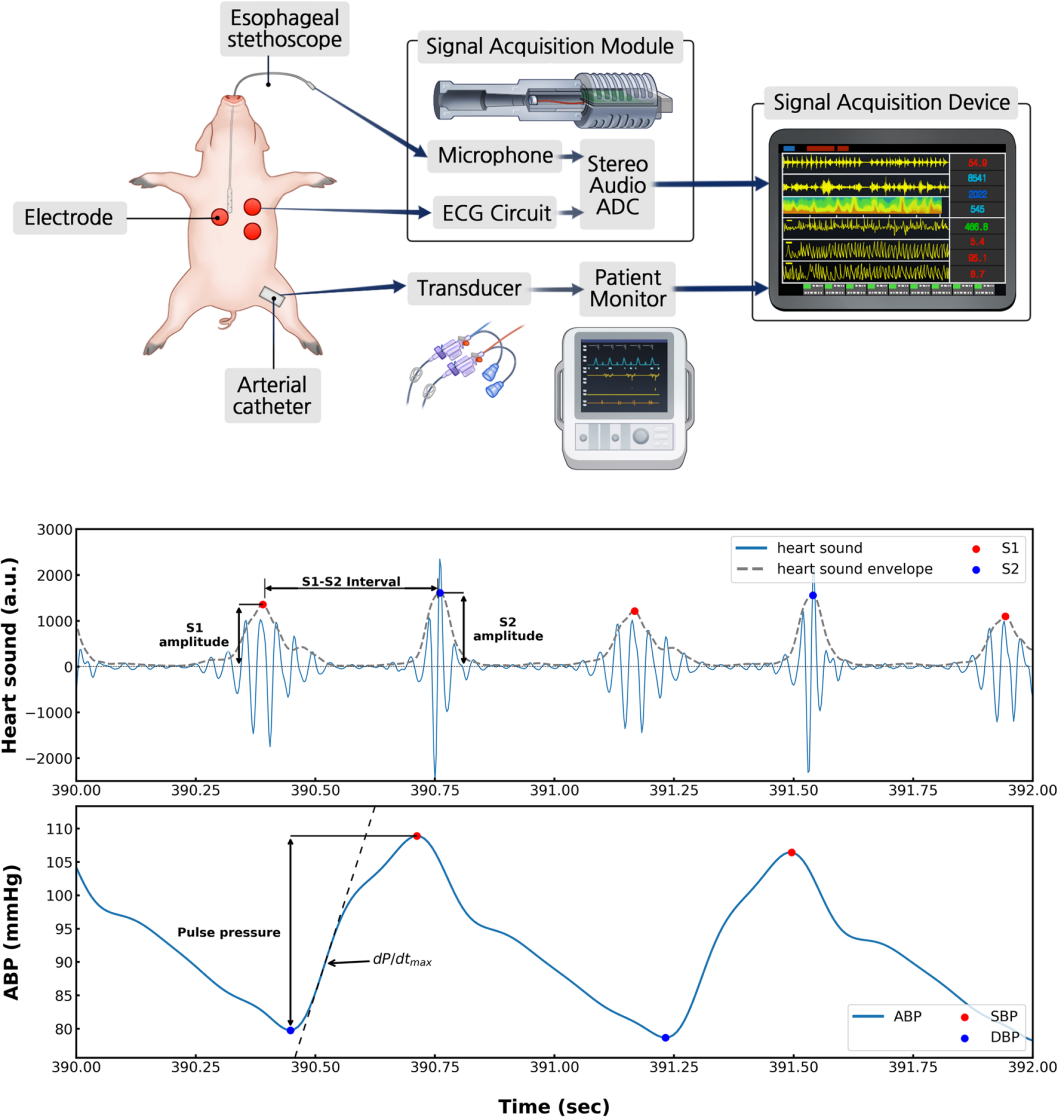
Schematic illustration of the esophageal stethoscope system and heart sound signal processing process. ECG, electrocardiography; ADC, analog-to-digital converter; ABP, arterial blood pressure; S1, first heart sound; S2, second heart sound; SBP, systolic blood pressure; DBP, diastolic blood pressure.

We measured three biosignals: the heart sounds, ABP, and electrocardiogram (ECG). The heart sounds were measured as sound signals with an electret microphone connected to an ESS inserted through the esophagus near the heart. The ECG was measured from lead II electrodes. The heart sounds and ECG were converted into audio signals using a stereo sound analog-to-digital converter (ADC) and stored in an Android app. The ABP was measured using a patient monitor from an arterial catheter inserted into the femoral artery via a pressure transducer. Finally, all the signals were collected and recorded simultaneously using SignalTAB (Signal House Co., Ltd., Seoul, Korea)^11^. It is an Android application that can record multiple biosignal waveforms simultaneously (Fig. 1).

To extract the heart sound features, we first applied a band-pass filter from 25 to 100 Hz using a second-order Chebyshev II FIR filter to separate the heart sound from the lung sounds. The envelope of the heart sound signal was calculated by squaring the absolute value of the filtered signal and using the Hilbert transform^12^. We detected the first heart sound (S1) and second heart sound (S2) in every cardiac cycle. These were defined using a temporal relationship with the R peak of the synchronized ECG signal: Two peaks were extracted in each cardiac cycle. The first and second peak points were defined as S1 and S2, respectively. Their heights were defined as S1 amplitude (S1amp) and S2 amplitude (S2amp), respectively. The R peaks of the ECG signal were extracted using a simple peak detection algorithm (peak_detection function in the SciPy python package) with manually calibrated thresholds for each recording. To extract the ABP features (the systolic blood pressure (SBP) and diastolic blood pressure (DBP) points), we used the feature extraction algorithm proposed by Sun et al.^13^. It uses the empirically defined threshold with a rule-based algorithm. The PP was calculated as the difference between SBP and DBP. dP/dt_max_ was calculated as the maximum value of the first derivative of the ABP. Both the values were obtained for each cardiac cycle. Finally, a sliding window approach was used with a window size of 10 s and a 5 s overlap to smoothing data. Each index was evaluated using 10-s segments of data to assess variations in haemodynamic and cardiac indices between pre- and post-intervention phases. Correlations between changes in haemodynamic and cardiac indices were assessed using data collected during the intervention periods. The signal processing analyses were performed using Python (Python Software Foundation, https://www.python.org).

### Statistical analysis

The hemodynamic and heart sound signal data were expressed as mean ± standard deviation. We assessed normality using the Shapiro–Wilk test. All the statistical measures were analyzed using one way ANOVA with repeated measures, and post-hoc comparisons were conducted using pairwise t-tests with Bonferroni’s multiple comparison. If Mauchley's test for sphericity showed that the assumption of sphericity was violated, we applied a greenhouse-gas correction to adjust the p-value. The correlation between variables was evaluated using Pearson correlation coefficients. We interpreted the correlation coefficients on the scatter plots as follows: a coefficient of < 0.1 indicates a negligible relationship, while > 0.9 indicates a very strong relationship, while values between 0.70 and 0.89 indicate a strong correlation, while values between 0.40 and 0.69 indicate a weak correlation, and those between 0.10 and 0.39 indicate a weak correlation^14^. The statistical analyses were performed using Python.

## Results

### Hemodynamic and heart sound variations between pre- and post-intervention

**Figure 2:**
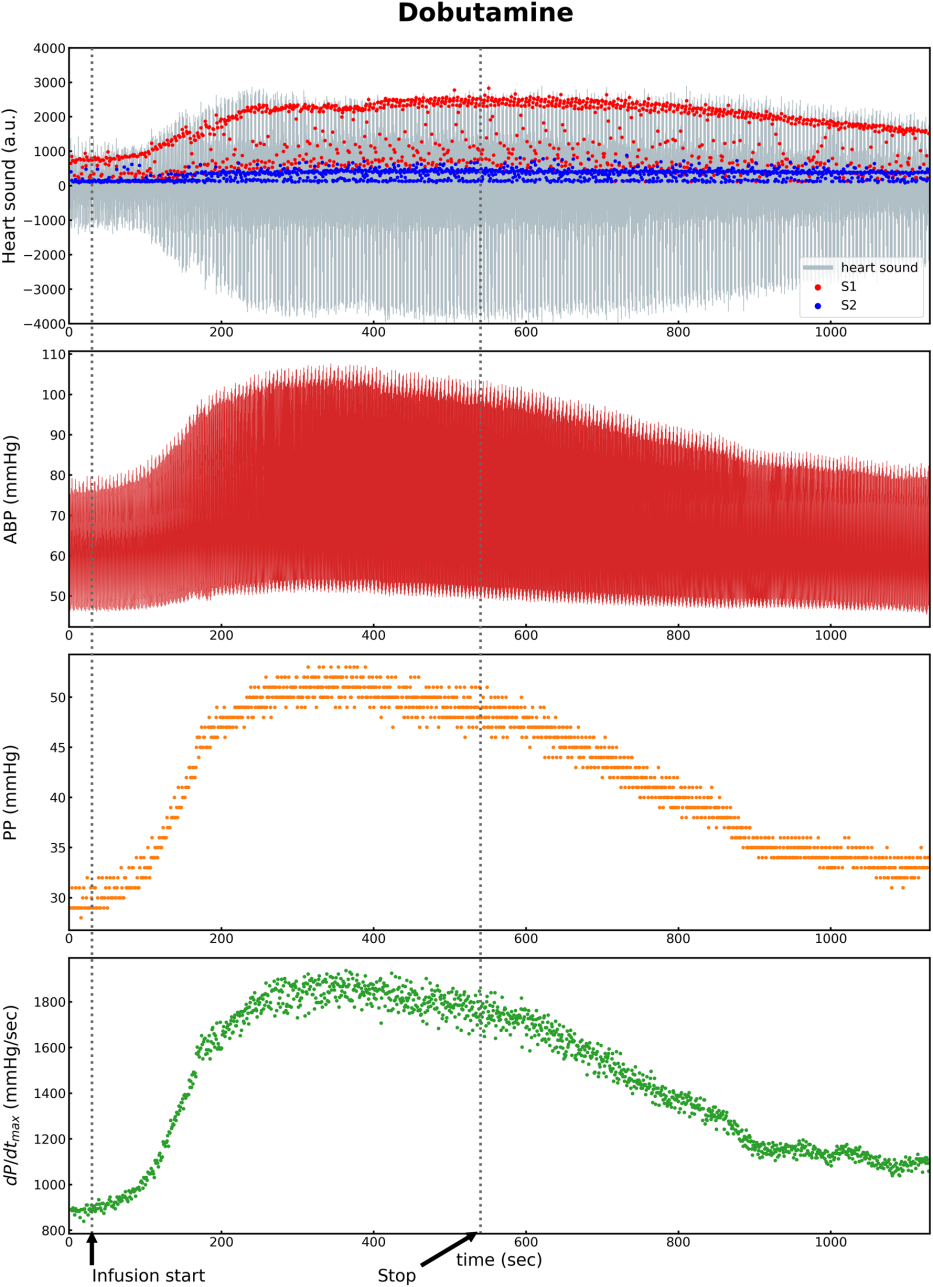
^11^. ABP, arterial blood pressure; PP, pulse pressure; dP/dt_max_, peak rate of increase in arterial pressure; S1, first heart sound; S2, second heart sound.

**Figure 3:**
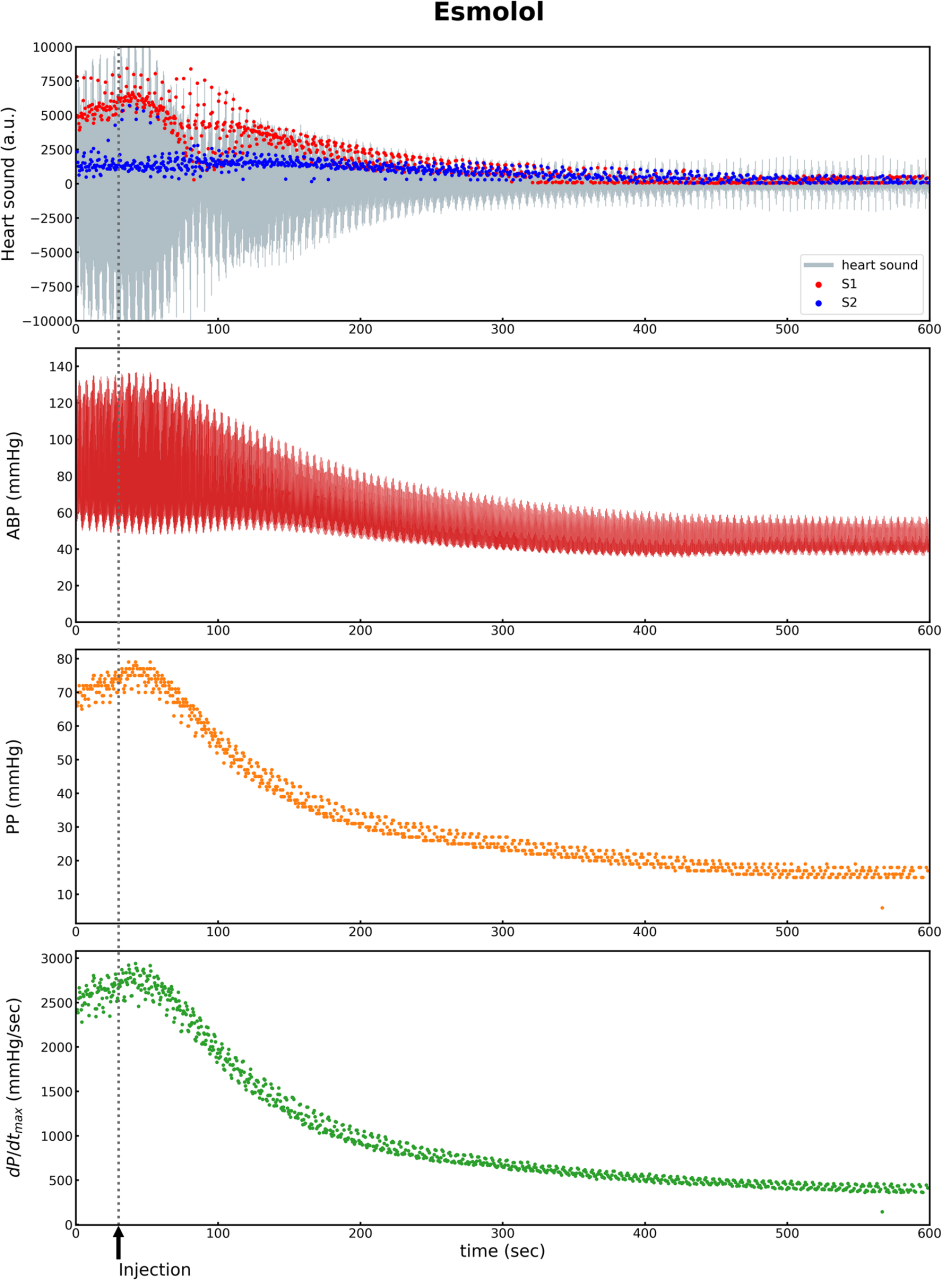
Representative plot of variations in hemodynamic status and heart sounds by esmolol administration. ABP, arterial blood pressure; PP, pulse pressure; dP/dt_max_, peak rate of increase in arterial pressure; S1, first heart sound; S2, second heart sound.

**Figure 4:**
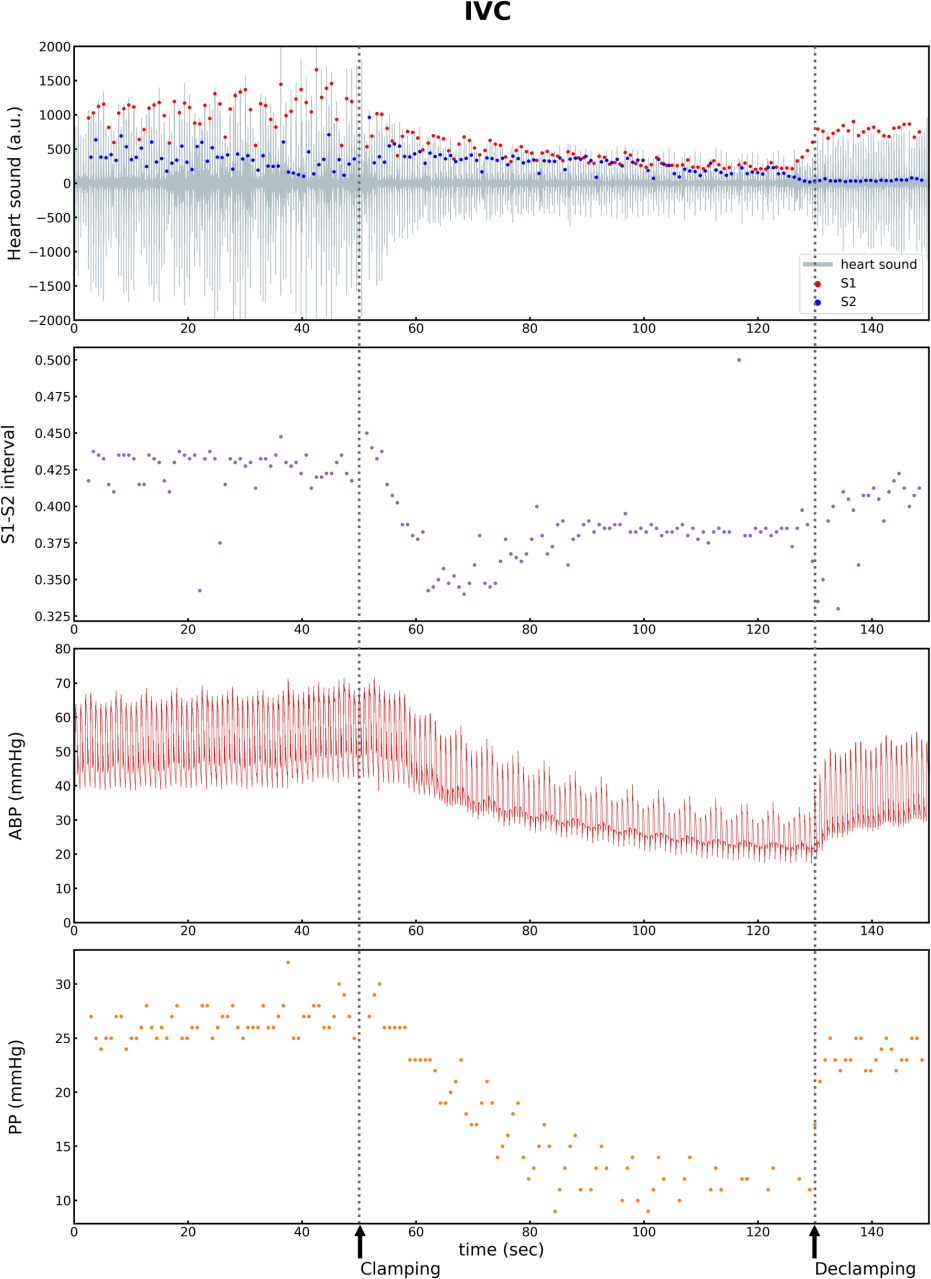
Representative plot of variations in hemodynamic status and heart sounds by IVC clamping. IVC, inferior vena cava; ABP, arterial blood pressure; PP, pulse pressure; S1, first heart sound; S2, second heart sound.

Of the 10 subjects, two were excluded owing to the poor heart sound signal. The data of the remaining eight subjects were included in the final analysis. Different hemodynamic variations were observed pre- and post-intervention depending on the type of drug (Table 1). After dobutamine injection, the SBP, PP, and dP/dt _max_ increased significantly owing to the increased myocardial contractility (ΔSBP 39.9 ± 11.1 mmHg: *F*(1,7) = 103.0, *p* < 0.001; ΔPP 19.8 ± 6.0 mmHg: *F*(1,7) = 88.6, *p* < 0.001; ΔdP/dt_max_ 932.8 ± 280.9: *F*(1,7) = −9.4, *p* < 0.001). Simultaneously, S1amp increased significantly (ΔS1amp 2765.7 ± 1291.8: *F*(1,7) = 36.7, *p* < 0.001) (Table 1) (Fig. 2). After esmolol injection, the SBP, PP, and dP/dt_max_ decreased owing to the decreased contractility (ΔSBP −30.3 ± 23.8 mmHg: *F*(1,7) = 13.2, *p* = 0.008; ΔPP −15.1 ± 20.4 mmHg: *F*(1,7) = 4.4, *p* = 0.074; ΔdP/dt _max_ −626.1 ± 740.9: *F*(1,7) = 5.7, *p* = 0.048) (Table 1). S1amp also decreased (ΔS1amp −1316.0 ± 1672.1: *F*(1,7) = 5.0, *p* = 0.061) (Table 1) (Fig. 3). After phenylephrine injection, the SBP and PP increased significantly owing to the increase in vascular resistance (ΔSBP 23.8 ± 12.5 mmHg; ΔPP 8.8 ± 8.3 mmHg), whereas the variations in the heart sounds were not significant. For nicardipine, the variation in the SBP was −21.2 ± 16.1 mmHg (Table 1). It was statistically significant (*p* = 0.008). Meanwhile, the variations in the heart sounds were not significant (Table 1). After IVC clamping, the SBP, PP, and S1amp decreased rapidly, and the PPV increased rapidly (Table 2). After IVC declamping, the hemodynamic and cardiac variables returned to the pre-clamping levels (Table 2 and Fig. 4).

**Table 1:** Variations in hemodynamic and heart sound indices by administration.

	Baseline	Dobutamine	Esmolol	Phenylephrine	Nicardipine
Hemodynamic variations					
ΔSBP (mmHg)	94.0 ± 14.4	39.9 ± 11.1***	−30.3 ± 23.6**	23.8 ± 12.5*	−21.2 ± 16.1*
ΔHeart Rate (bpm)	70.5 ± 12.6	51.4 ± 13.7***	−24.9 ± 19.2**	−3.0 ± 10.8	−0.3 ± 6.2
ΔPulse Pressure (mmHg)	33.5 ± 7.8	19.9 ± 6.0***	−15.1 ± 20.4	8.8 ± 8.3*	−4.5 ± 6.7
ΔPulse Pressure Variation	9.6 ± 2.9	2.0 ± 3.5	0.2 ± 5.5	0.6 ± 2.1	2.3 ± 2.8
ΔdP/dt_max_	733.6 ± 205.4	932.8 ± 280.9***	−626.1 ± 740.9*	135.6 ± 305.4	−30.1 ± 248.0
Heart Sound variations					
ΔS1 amplitude (AU)	668.6 ± 312.5	2765.7 ± 1291.8***	−1316.0 ± 1672.1	61.8 ± 414.1	−90.8 ± 259.8
ΔS2 amplitude (AU)	827.6 ± 515.5	618.4 ± 794.7*	−382.8 ± 315.4*	55.0 ± 145.3	−199.1 ± 169.5*
ΔS1–S2 interval (ms)	300.4 ± 86.9	−36.6 ± 106.5	−2.5 ± 57.5	−3.7 ± 33.5	3.4 ± 20.1
ΔS1–S2 interval variation	32.1 ± 25.9	5.3 ± 31.1	6.5 ± 23.6	−5.4 ± 16.5	0.3 ± 5.8

Δ, difference between the values before and after intervention; SBP, systolic blood pressure; dP/dt_max_, peak rate of increase in arterial pressure; AU, arbitrary unit.

*p < 0.05; **p < 0.01; ***p < 0.001: *p*-value comparing pre- and post-intervention variables using one-way ANOVA with repeated measures.

**Table 2:** Variations in hemodynamic and heart sound indices by IVC clamping.

	Before clamping (Baseline)	During clamping	After declamping
Hemodynamic variables			
Systolic Blood Pressure (mmHg)	91.0 ± 14.4	61.1 ± 11.7**	86.5 ± 12.6**
Heart Rate (bpm)	83.5 ± 13.2	83.8 ± 12.0	82.6 ± 11.5
Pulse Pressure (mmHg)	39.2 ± 9.8**	22.7 ± 8.2***	34.6 ± 10.2**
Pulse Pressure Variation	11.8 ± 3.6	26.8 ± 10.1**	11.2 ± 4.7**
dP/dt_max_	900.8 ± 352.4	510.8 ± 154.0*	768.9 ± 284.7
Heart sound variables			
S1 amplitude (AU)	862.3 ± 281.8	474.8 ± 157.6*	732.2 ± 295.2
S2 amplitude (AU)	735.3 ± 463.8	431.6 ± 251.6	686.5 ± 486.4
ΔS1–S2 interval (ms)	327.9 ± 53.0	310.5 ± 47.4	325.6 ± 54.0
ΔS1–S2 interval variation	25.9 ± 20.4	29.4 ± 16.6	29.1 ± 21.3

dP/dt_max_, peak rate of increase in arterial pressure; AU, arbitrary unit.

*p < 0.05; **p < 0.01; ***p < 0.001: *p*-value compared with the previous step by post hoc paired t-tests with Bonferroni’s multiple comparisons.

### Correlation between hemodynamic changes and changes in heart sound indices

**Figure 5:**
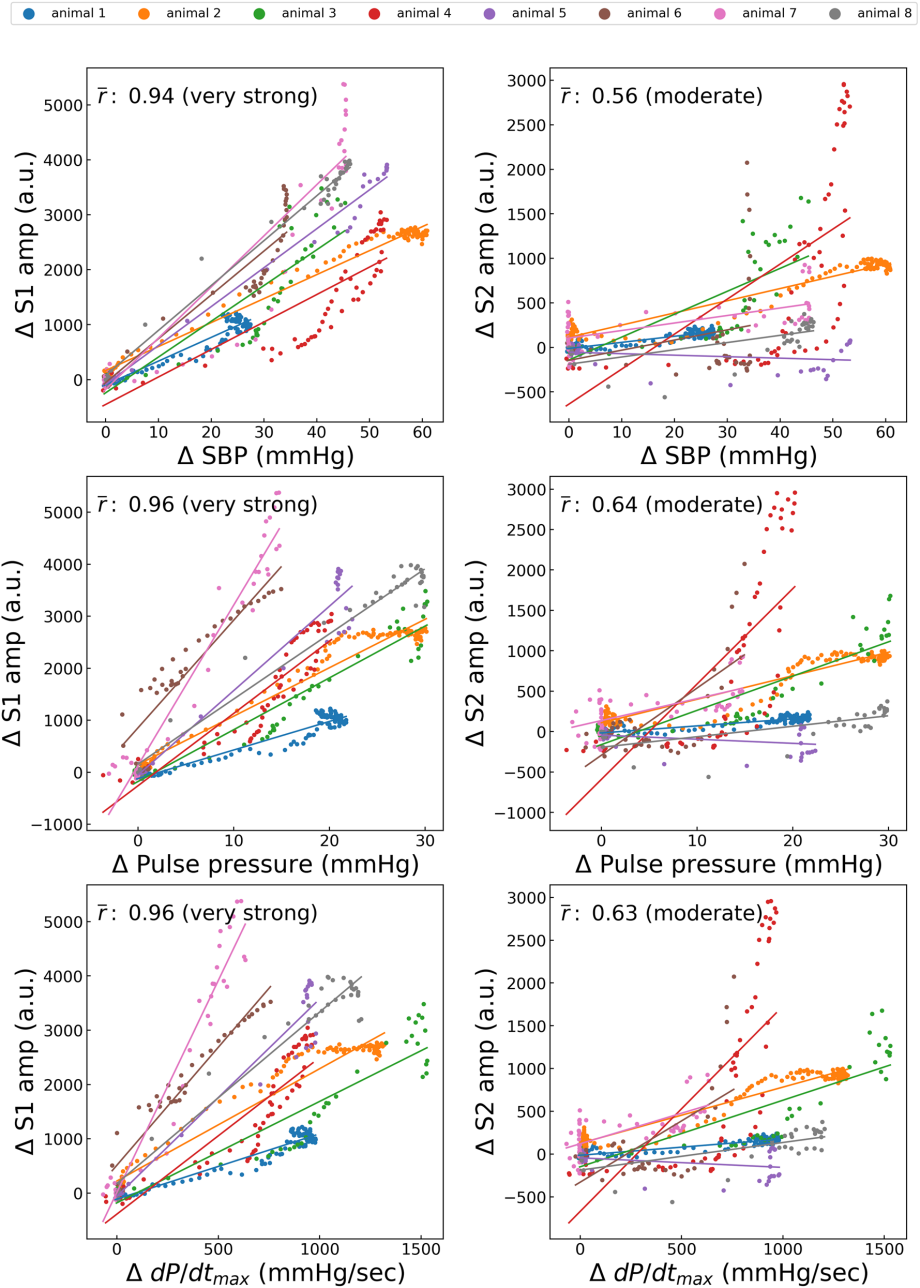
Correlations between dobutamine-induced variations in hemodynamic status and heart sound index. S1amp, amplitude of the first heart sound; S2amp, amplitude of the second heart sound; SBP, systolic blood pressure; dP/dt_max_, peak rate of increase in arterial pressure; *r̄*, average of the correlation coefficient in each case. Each point represents the result of a 10-s window with a sliding window overlapping by 5 s. The average number of points per animal is 63.0.

**Figure 6:**
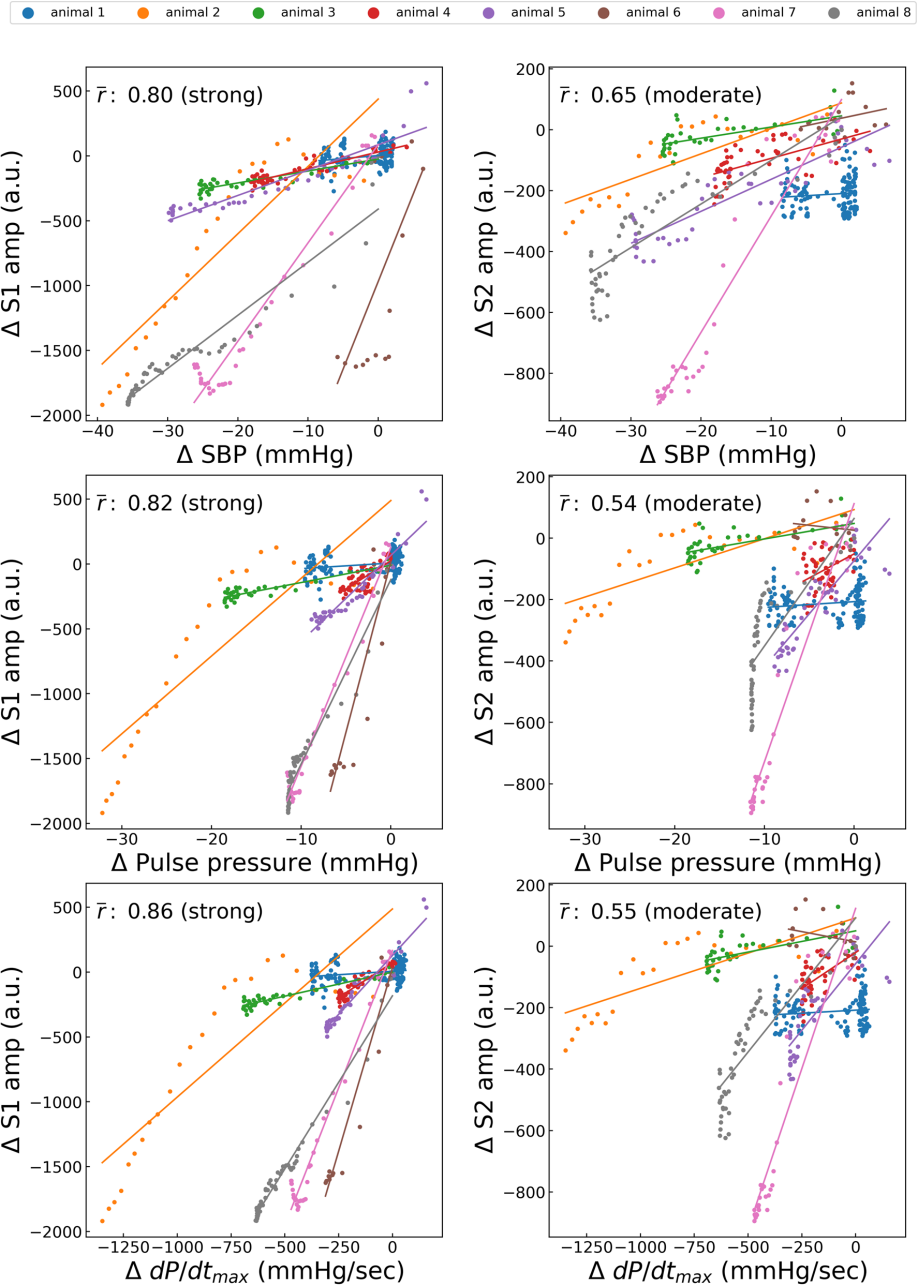
Correlations between esmolol-induced variations in hemodynamic status and heart sound index. S1amp, amplitude of the first heart sound; S2amp, amplitude of the second heart sound; SBP, systolic blood pressure; dP/dt_max_, peak rate of increase in arterial pressure; *r̄*, average of the correlation coefficient in each case. Each point represents the result of a 10-s window with a sliding window overlapping by 5 s. The average number of points per animal is 49.0.

**Figure 7:**
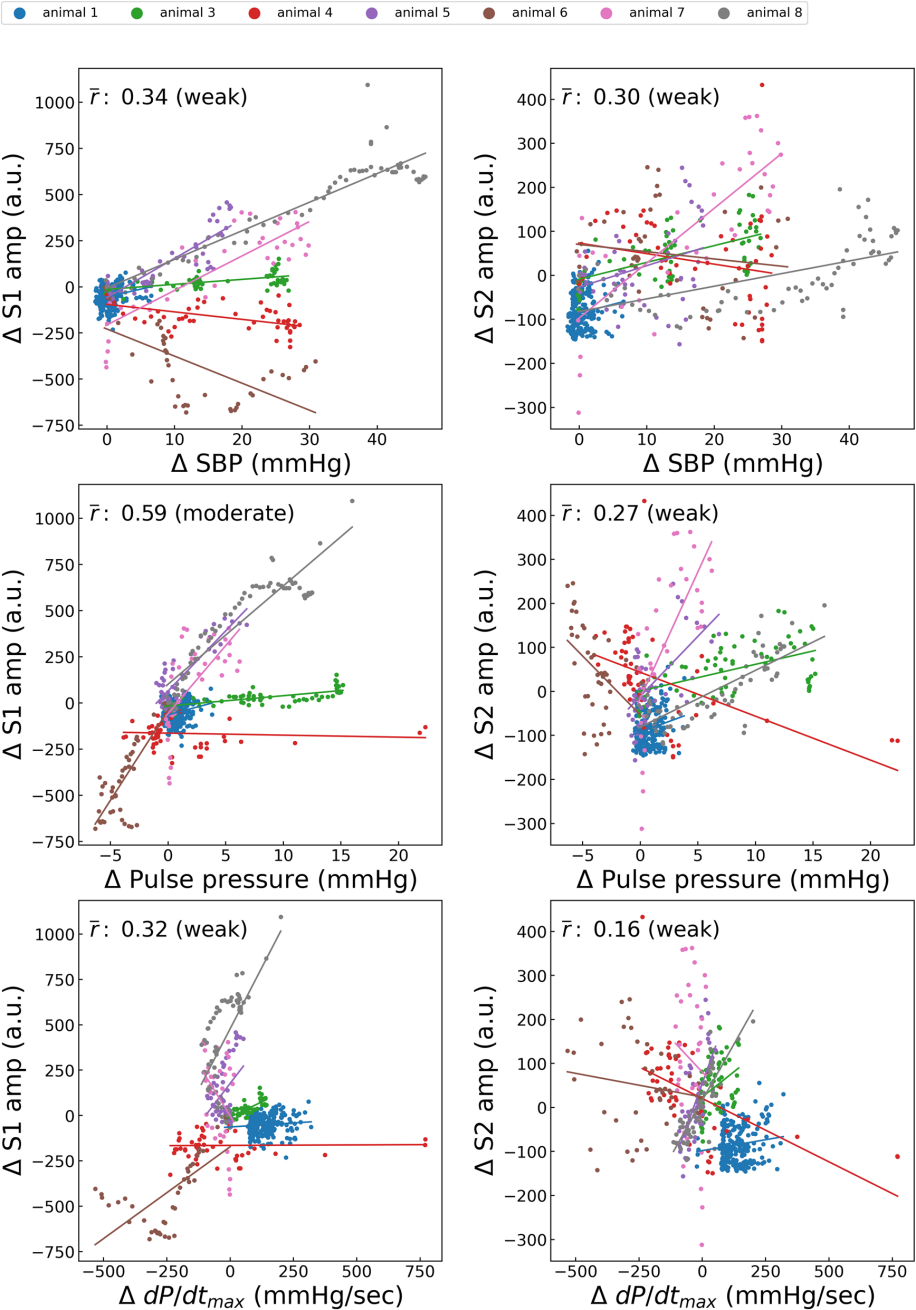
Correlations between phenylephrine-induced variations in hemodynamic status and heart sound index. S1amp, amplitude of the first heart sound; S2amp, amplitude of the second heart sound; SBP, systolic blood pressure; dP/dt_max_, peak rate of increase in arterial pressure; *r̄*, average of the correlation coefficient in each case. Each point represents the result of a 10-s window with a sliding window overlapping by 5 s. The average number of points per animal is 91.5.

**Figure 8:**
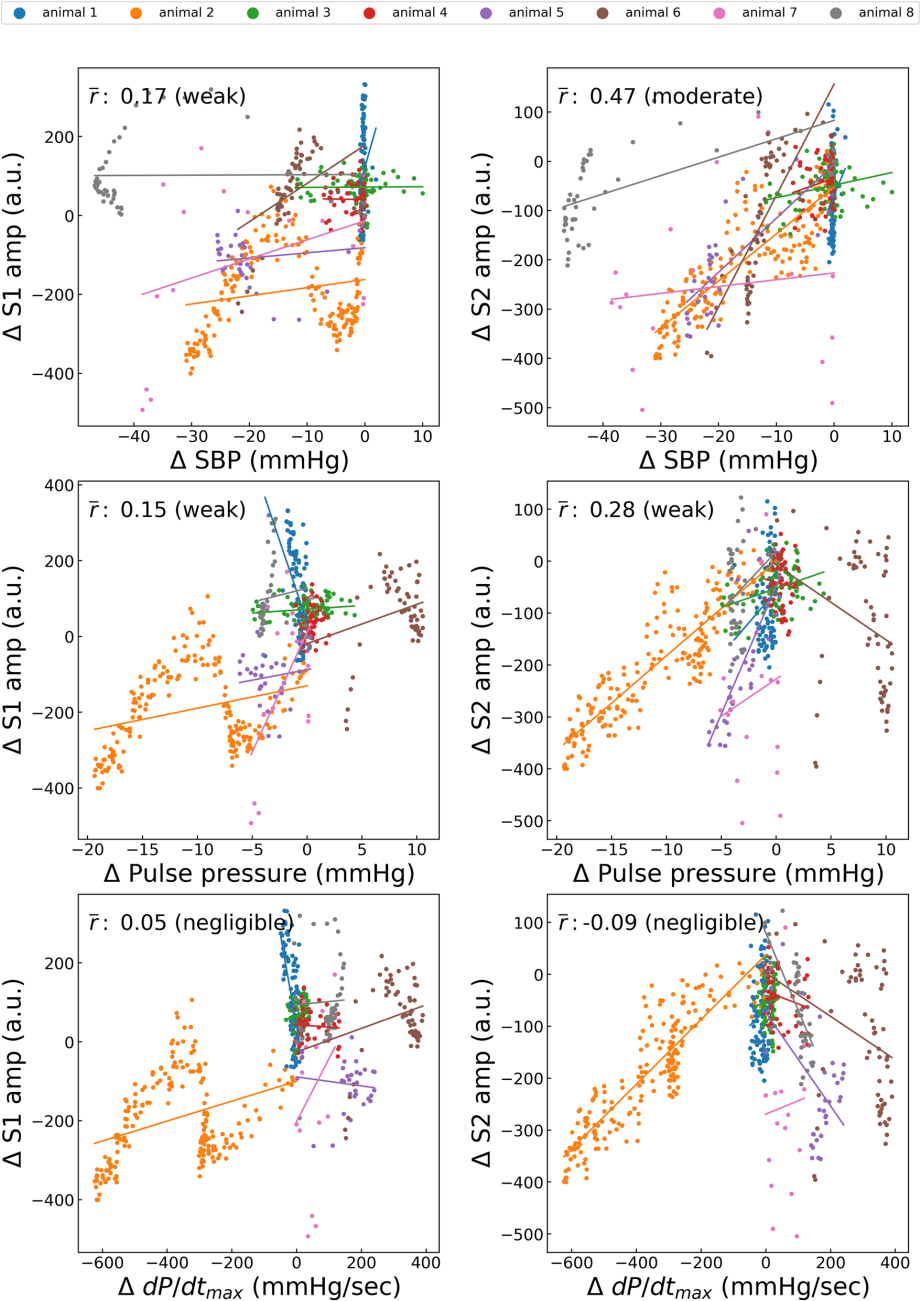
Correlations between nicardipine-induced variations in hemodynamic status and heart sound index. S1amp, amplitude of the first heart sound; S2amp, amplitude of the second heart sound; SBP, systolic blood pressure; dP/dt_max_, peak rate of increase in arterial pressure; *r̄*, average of the correlation coefficient in each case. Each point represents the result of a 10-s window with a sliding window overlapping by 5 s. The average number of points per animal is 72.3.

**Figure 9:**
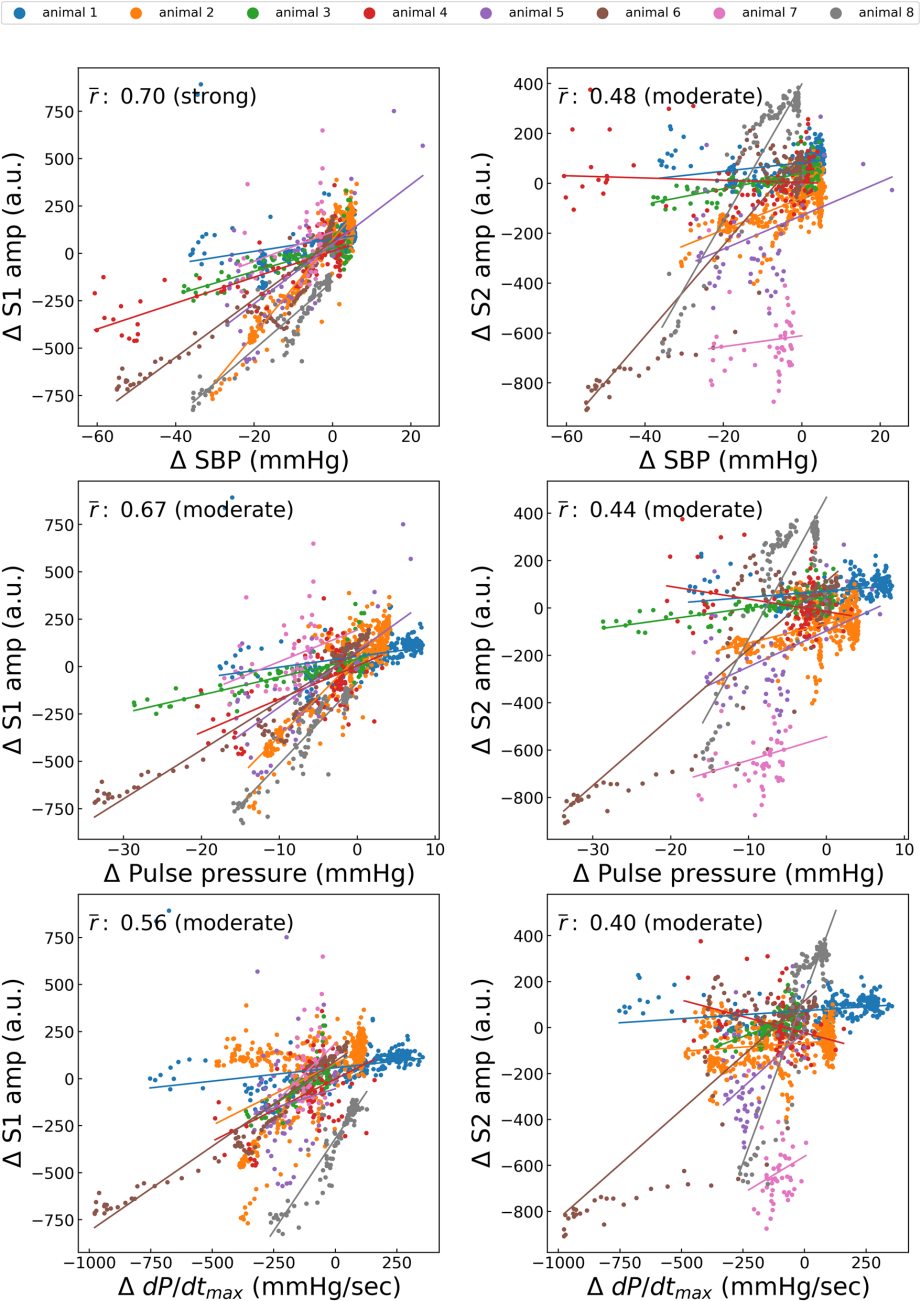
Correlations between variations in hemodynamic status owing to IVC clamping and heart sound index. S1amp, amplitude of the first heart sound; S2amp, amplitude of the second heart sound; SBP, systolic blood pressure; dP/dt_max_, peak rate of increase in arterial pressure; *r̄*, average of the correlation coefficient in each case. Each point represents the result of a 10-s window with a sliding window overlapping by 5 s. The average number of points per animal is 157.6.

Changes The variations in ΔS1amp owing to dobutamine administration showed a good positive correlation with those in the SBP, PP, and dP/dt_max_. The mean values of the correlation coefficients of ΔS1amp with ΔSBP, ΔPP, and ΔdP/dt_max_ during dobutamine administration were 0.94, 0.96, and 0.96, respectively (Fig. 5). Changes in ΔS1amp owing to esmolol administration also showed a good positive correlation with those in the SBP, PP, and dP/dt_max_. The mean values of the correlation coefficients of ΔS1amp with ΔSBP, ΔPP, and ΔdP/dt during esmolol administration were 0.80, 0.82, and 0.86, respectively (Fig. 6). However, the hemodynamic changes caused by phenylephrine and nicardipine administration did not correlate significantly with any changes in S1amp or S2amp. The mean correlation coefficients ranged between −0.09 and 0.59 for all the cases (Figs. 7 and 8). Changes in SBP and PP caused by IVC clamping were positively correlated with those in S1amp. The mean Pearson coefficients were 0.70 and 0.67, respectively (Fig. 9).

## Discussion

In the current study, we identified the changes in hemodynamic parameters induced by different mechanisms and their correlation with the heart sound index. In particular, we observed a strong correlation between changes in S1amp and those in the hemodynamic parameters caused by changes in myocardial contractility. Meanwhile, the variations in the hemodynamic parameters owing to those in the vascular resistance were not consistently correlated with changes in heart sound indices.

The primary component of the heart sound is the vibration caused by the abrupt acceleration or deceleration of blood within the cardiovascular system^16^. Heart sounds are broadly categorized into S1 and S2: S1 has a slightly lower pitch and occurs when the atrioventricular valves close. Meanwhile, S2 has a higher pitch and is produced by the closure of the arterial valves^17,18^. In our previous work, we observed that S1amp increased in cases where ephedrine was administered^7^. Ephedrine is a drug derived from the ephedra plant. It has a predominant β-adrenergic effect with additional vasoconstrictor activity^19,20^. Cases in which ephedrine was injected were analyzed in the previous study because it is commonly administered to treat hypotension during anesthesia, making it easier to collect data from electronic medical record. However, it can be difficult to observe the effect of pure variations in myocardial contractility. Meanwhile, dobutamine is a direct-acting agent that acts on the β_1_-adrenoreceptor. This makes it more suitable for determining the effectiveness of a pure increase in contractility^21^. In this experiment, dobutamine administration successfully produced a significant increase in cardiac output owing to a large increase in contractility. It resulted in a strongly correlated variation in S1amp. Because the pressure from the contraction of the ventricle is the primary force that closes the atrioventricular valve, this provides an explanation for why variations in the myocardial contractile force alters S1amp and increases the potential of S1 as a monitoring modality specific to cardiac contractility.

However, hemodynamic variations with changes in the systemic vascular resistance induced by the administration of phenylephrine and nicardipine did not display a correlation with the heart sound parameters in our study. This was inconsistent with our anticipation based on our previous research, wherein the administration of phenylephrine and nicardipine significantly affected S2amp. A likely explanation for this is that the correlation between the vascular resistance and S2amp is weaker than that between myocardial contractility and S1amp. In the previous study, the correlation between S2amp and SVR (*r*^2^ = 0.285) was relatively weak compared with that between S1amp and dP/dt (*r*^2^ = 0.679), although the correlation is described as statistically significant^7^. Therefore, it is likely that the variations in vascular resistance produced by the administration of phenylephrine and nicardipine in this experiment were insufficient to produce significant variations in the heart sounds. If this is true, it indicates that the variations in vascular resistance that typically occur in surgical patients do not produce detectable variations in the heart sounds. Therefore, it may be ineffective to monitor the variations in the vascular resistance by heart sound measurements. Another explanation is that the anatomical or physiological differences between humans and pigs caused these disparities. The porcine organ resembles a conventional “valentine heart”. It is influenced by its position within the thorax and the pig's unguligrade stance. By contrast, the human heart has a trapezoidal silhouette, which reflects our upright posture^22^. Another report revealed that the diameter of the great vessels including the ascending aorta and main pulmonary artery is smaller in pigs than in humans^23^. This is important because we acquire heart sounds by inserting the ESS through the esophagus and receiving sound signals through it. Therefore, these anatomical variations and differences in the positioning of the ESS relative to the heart and great vessels could have affected the shape and amplitude of the acquired heart sound signal. The difference may also have been owing to the condition of the subjects. In this study, the drug was administered to animals with normal vital signs, whereas it was administered to patients with abnormal vital signs in previous studies. This was because the patients were given the drug only to correct certain abnormal vital conditions, which may have contributed to the higher effect of S2amp.

In the hemodynamic variations owing to IVC clamping, the variations in S1amp were correlated more strongly with those in the SBP and PP compared with those in dP/dt_max_. IVC clamping is a technique that can reduce bleeding by decreasing the central venous pressure during liver resection. However, it is known to cause intraoperative hemodynamic instability owing to the reduced venous return^24–26^. Dobutamine yielded a correlation between S1amp and dP/dt_max_ that was similar to or marginally stronger than that between S1amp and PP. Meanwhile, IVC clamping caused a rapid reduction in the cardiac output through venous return restriction without a significant effect on the contractility. This resulted in a relatively weak correlation between S1amp and dP/dt_max_. Although the arterial dP/dt_max_ is influenced by various central and peripheral arterial factors, the radial or femoral dP/dt_max_ is known to provide a good tracking of the variations in the left ventricular contractility as loading and inotropic conditions are altered^27^. Therefore, this phenomenon (the relatively weak correlation of dP/dtmax with S1amp) is likely to reflect the mechanism of action of IVC clamping on the hemodynamic variations (reduction in the cardiac output not attributable to myocardial contractility). Given these correlations, it is likely that the cause of hemodynamic variations can be differentiated (i.e., determine whether these are owing to a variation in myocardial contractility or other causes) by analyzing the heart sounds.

This study has several limitations. First, as noted above, we did not observe a consistent correlation between the variations in S2amp and those in the vascular resistance. This result either reflects a weak or zero correlation between the variables or is caused by the physiological differences between humans and pigs. In particular, the inability to directly measure vascular resistance to determine the threshold for the effects of phenylephrine and nicardipine may have been a major contributor to these limitations. Future studies that employ real-time vascular resistance monitoring via intracardiac catheters may be able to address these limitations. However, further research is required to determine the actual cause. Second, although the intra-individual correlations were strong, the inter-individual variability was significantly large so that generalized relationships between the cardiac and hemodynamic variables could not be established. Compared with the correlations between the variables in each case, those between the variables in all the cases combined were weak (Supplementary Figs. 1, 2, and 3 and Supplementary Tables 1, 2, 3, 4, 5, and 6). In addition to this inter-individual variation, the unavailability of an absolute baseline value for the heart sound hinders its use as a monitoring indicator. Therefore, to develop this as a practical monitoring tool, it should be a means to use the trend based on the initial baseline value. Third, owing to the rapid decrease in cardiac output during IVC clamping, the heart sound signal became significantly weak. This, in turn, resulted in a low peak detection performance and failure to measure the respiratory variation of beat-to-beat heart sound. That is, the objective of identifying a non-invasive alternative to fluid responsiveness monitoring was not achieved. In future studies, partial or gradual clamping of the IVC or fluid loading to drive gradual, incremental variations in the cardiac output may help overcome this limitation. Fourth, data from two subjects were excluded owing to the inferior heart sound signals. The equipment we used to acquire the heart sounds comprised an early Android-based software on a tablet computer. It could not examine the signal acquisition status during the experiment. However, it was necessary for it to examine the signal sent to the server after the experiment was completed. Currently, with the update, it is feasible to observe the signal in real-time on the tablet as it is being acquired. Therefore, we consider that this problem would not occur in future clinical trials. Fifth, a cardiac pressure–volume loop analysis could be performed to determine the end-systolic pressure–volume relationship (ESPVR) and end-diastolic pressure–volume relationship (EDPVR). These reflect the actual myocardial contractility and ventricular compliance, respectively. However, the analysis was not performed in this experiment. Finally, only male animals were used as the experimental subjects, which may cause sex bias in the interpretation and application of the experimental results^28^.

## Conclusion

To conclude, the hemodynamic variations related to myocardial contractility were significantly correlated with the variations in the S1 amplitude. However, the variations related to the vascular resistance did not correlate with those in the heart sound parameters. This indicates that heart sounds can be used to monitor variations in myocardial contractility and the cardiac output. Moreover, these may be a means to differentiate the cause of hemodynamic variations.

### Supplementary Information

Supplementary Information 1.

Supplementary Information 2.

## Data Availability

The datasets used and/or analyzed during the current study available from the corresponding author on reasonable request.

## Code availability

The core biosignals processing code is available from https://github.com/baporlab/PigHemoHeartsound^15^.
