# Supplementary material for: Correlations between heart sound components and hemodynamic variables
Source: Sci Rep. 2024 Apr 13;14:8602. doi: 10.1038/s41598-024-59362-3 (PMC11016121; doi:10.1038/s41598-024-59362-3)

**Supplementary Figure 1.** Correlations between dobutamine-induced variations in hemodynamic status and heart sound index. The single regression lines plotted summarize the variations in all the cases. S1amp, amplitude of the first heart sound; S2amp, amplitude of the second heart sound; SBP, systolic blood pressure; dP/dt_max_, peak rate of increase in arterial pressure.

**
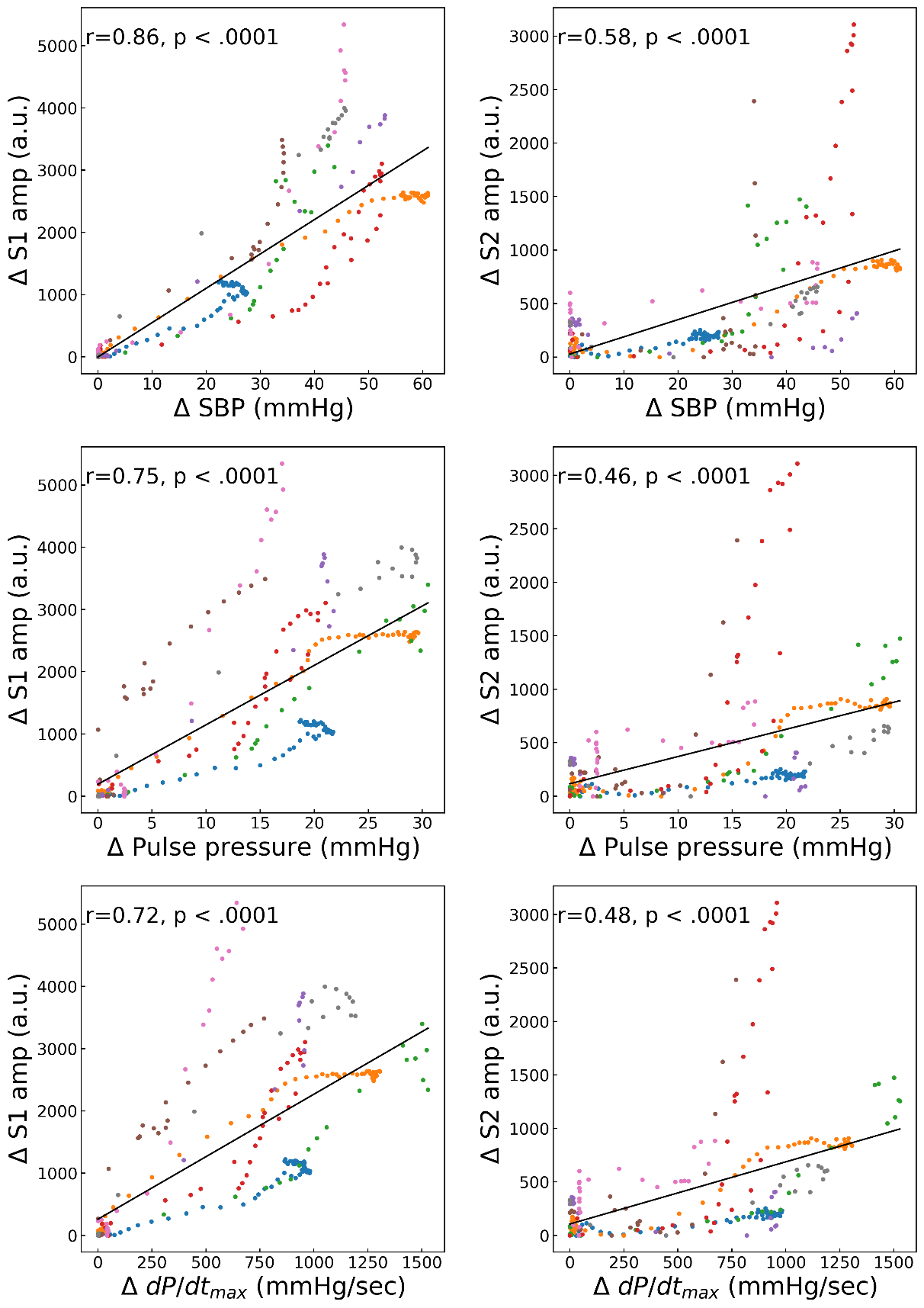
**

**Supplementary Figure 2.** Correlations between esmolol-induced variations in hemodynamic status and heart sound index. The single regression lines plotted summarize the variations in all the cases. S1amp, amplitude of the first heart sound; S2amp, amplitude of the second heart sound; SBP, systolic blood pressure; dP/dt_max_, peak rate of increase in arterial pressure.**
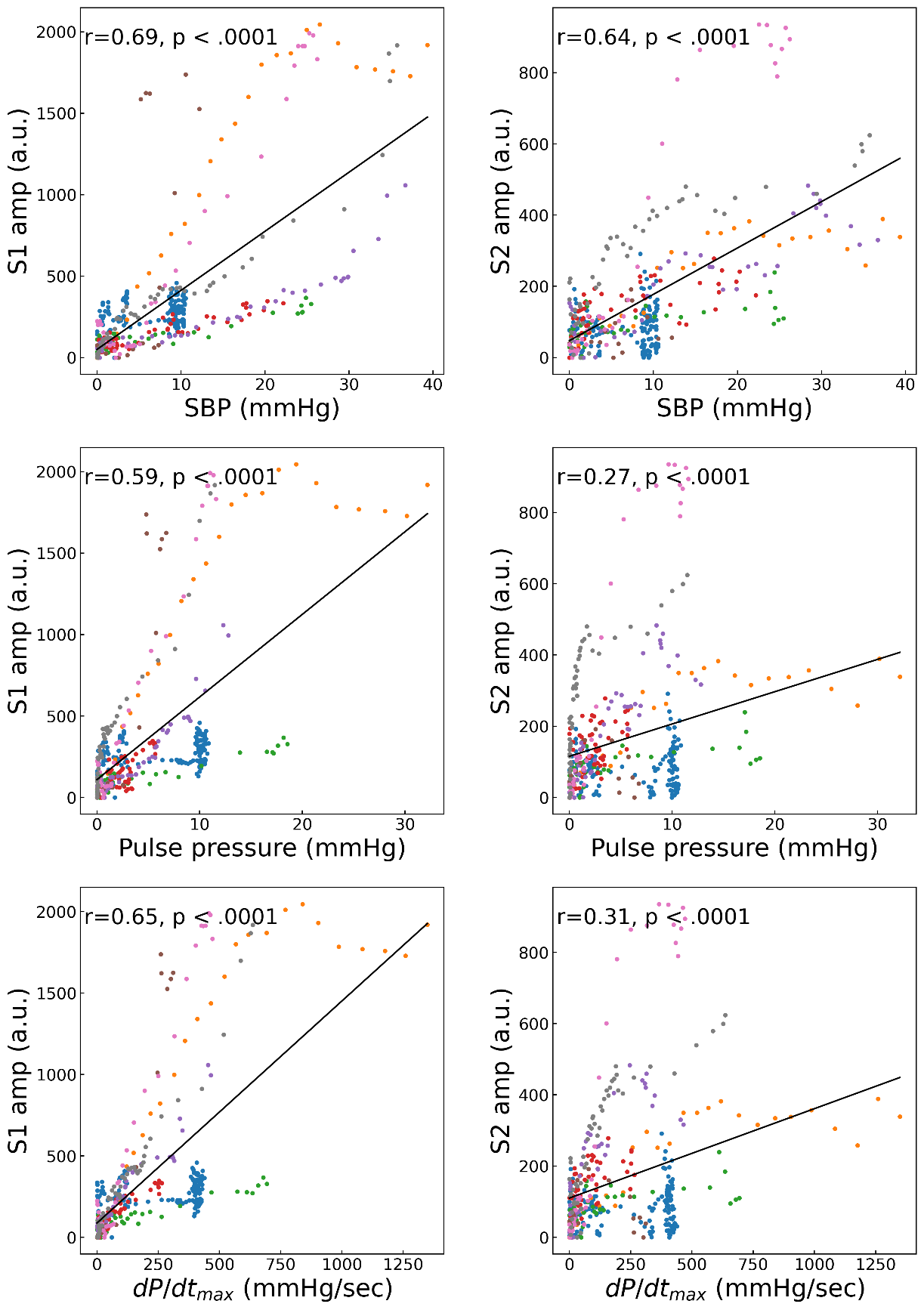
**

**Supplementary Figure 3.** Correlations between variations in hemodynamic status and heart sound index by inferior-vena-cava clamping. The single regression lines plotted summarize the variations in all the cases. S1amp, amplitude of the first heart sound; S2amp, amplitude of the second heart sound; SBP, systolic blood pressure; dP/dt_max_, peak rate of increase in arterial pressure.


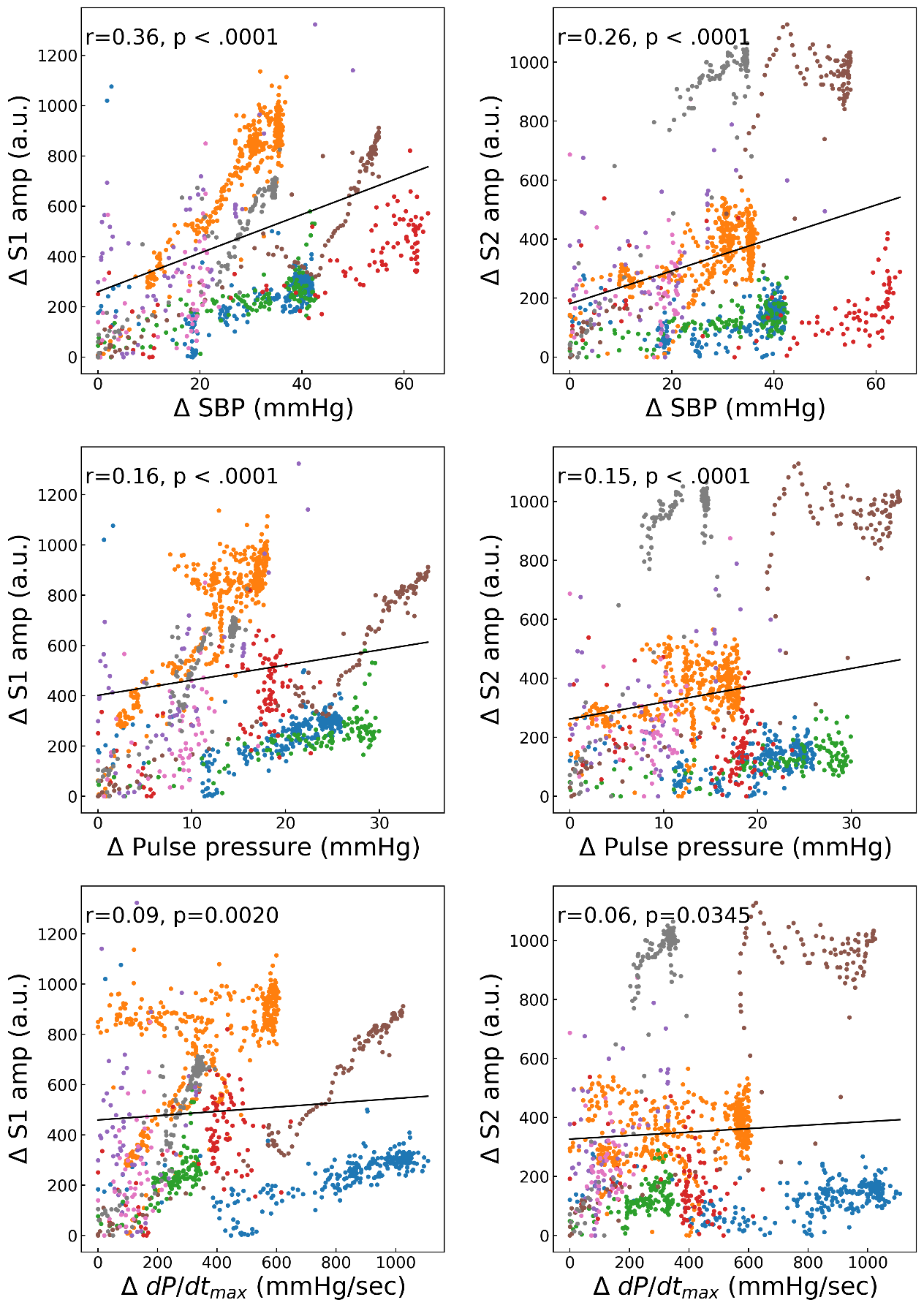

Supplement: Supplementary file 1 — Supplementary Information 1. [file 41598_2024_59362_MOESM1_ESM.docx]
