# Supplementary material for: Correlations between heart sound components and hemodynamic variables
Source: Sci Rep. 2024 Apr 13;14:8602. doi: 10.1038/s41598-024-59362-3 (PMC11016121; doi:10.1038/s41598-024-59362-3)
Supplement: Supplementary file 2 — Supplementary Information 2. [file 41598_2024_59362_MOESM2_ESM.docx]

**Supplementary Table 1.** Correlations between s1 amplitude and hemodynamic variables by dobutamine administration. Described for each case.

|  | **ΔS1amp and ΔSBP** | | **ΔS1amp and ΔPP** | | **ΔS1amp and ΔdP/dt_max_** | |
| --- | --- | --- | --- | --- | --- | --- |
| Case | r | *p*-value | r | *p*-value | r | *p*-value |
| 1 | 0.952 | < 0.001 | 0.957 | < 0.001 | 0.964 | < 0.001 |
| 2 | 0.995 | < 0.001 | 0.985 | < 0.001 | 0.981 | < 0.001 |
| 3 | 0.888 | < 0.001 | 0.963 | < 0.001 | 0.945 | < 0.001 |
| 4 | 0.855 | < 0.001 | 0.912 | < 0.001 | 0.894 | < 0.001 |
| 5 | 0.995 | < 0.001 | 0.979 | < 0.001 | 0.981 | < 0.001 |
| 6 | 0.909 | < 0.001 | 0.901 | < 0.001 | 0.961 | < 0.001 |
| 7 | 0.949 | < 0.001 | 0.978 | < 0.001 | 0.977 | < 0.001 |
| 8 | 0.990 | < 0.001 | 0.983 | < 0.001 | 0.975 | < 0.001 |

S1amp, amplitude of first heart sound; SBP, systolic blood pressure; dP/dt_max_, peak rate of increase in arterial pressure.

**Supplementary Table 2.** Correlations between s1 amplitude and hemodynamic variables by esmolol administration. Described for each case.

|  | **ΔS1amp and ΔSBP** | | **ΔS1amp and ΔPP** | | **ΔS1amp and ΔdP/dt_max_** | |
| --- | --- | --- | --- | --- | --- | --- |
| Case | R | *p*-value | r | *p*-value | r | *p*-value |
| 1 | 0.214 | 0.011 | 0.218 | 0.010 | 0.219 | 0.009 |
| 2^†^ | 0.900 | < 0.001 | 0.856 | < 0.001 | 0.863 | < 0.001 |
| 3 | 0.918 | < 0.001 | 0.930 | < 0.001 | 0.935 | < 0.001 |
| 4 | 0.916 | < 0.001 | 0.716 | < 0.001 | 0.918 | < 0.001 |
| 5 | 0.922 | < 0.001 | 0.951 | < 0.001 | 0.966 | < 0.001 |
| 6 | 0.226 | 0.115 | 0.641 | < 0.001 | 0.748 | < 0.001 |
| 7 | 0.979 | < 0.001 | 0.989 | < 0.001 | 0.988 | < 0.001 |
| 8 | 0.954 | < 0.001 | 0.967 | < 0.001 | 0.986 | < 0.001 |

S1amp, amplitude of first heart sound; SBP, systolic blood pressure; dP/dt_max_, peak rate of increase in arterial pressure.

**Supplementary Table 3.** Correlations between s1 amplitude and hemodynamic variables induced by inferior-vena-cava clamping. Described for each case.

|  | **ΔS1amp and ΔSBP** | | **ΔS1amp and ΔPP** | | **ΔS1amp and ΔdP/dt_max_** | |
| --- | --- | --- | --- | --- | --- | --- |
| Case | r | *p*-value | r | *p*-value | r | *p*-value |
| 1 | 0.307 | < 0.001 | 0.303 | < 0.001 | 0.322 | < 0.001 |
| 2 | 0.946 | < 0.001 | 0.878 | < 0.001 | 0.545 | < 0.001 |
| 3 | 0.743 | < 0.001 | 0.750 | < 0.001 | 0.721 | < 0.001 |
| 4 | 0.792 | < 0.001 | 0.585 | < 0.001 | 0.503 | < 0.001 |
| 5 | 0.661 | < 0.001 | 0.644 | < 0.001 | 0.159 | 0.243 |
| 6 | 0.935 | < 0.001 | 0.958 | < 0.001 | 0.973 | < 0.001 |
| 7 | 0.319 | 0.025 | 0.331 | 0.020 | 0.371 | 0.009 |
| 8 | 0.926 | < 0.001 | 0.933 | < 0.001 | 0.926 | < 0.001 |

S1amp, amplitude of first heart sound; SBP, systolic blood pressure; dP/dtmax, peak rate of increase in arterial pressure.

**Supplementary Table 4.** Correlations between s2 amplitude and hemodynamic variables by dobutamine administration. Described for each case.

|  | **ΔS2amp and ΔSBP** | | **ΔS2amp and ΔPP** | | **ΔS2amp and ΔdP/dt_max_** | |
| --- | --- | --- | --- | --- | --- | --- |
| Case | r | *p*-value | r | *p*-value | r | *p*-value |
| 1 | 0.867 | < 0.001 | 0.869 | < 0.001 | 0.873 | < 0.001 |
| 2 | 0.969 | < 0.001 | 0.956 | < 0.001 | 0.969 | < 0.001 |
| 3 | 0.768 | < 0.001 | 0.896 | < 0.001 | 0.861 | < 0.001 |
| 4 | 0.624 | < 0.001 | 0.718 | < 0.001 | 0.691 | < 0.001 |
| 5 | -0.277 | 0.088 | -0.359 | 0.025 | -0.354 | 0.027 |
| 6 | 0.252 | 0.127 | 0.701 | < 0.001 | 0.614 | < 0.001 |
| 7 | 0.648 | < 0.001 | 0.650 | < 0.001 | 0.675 | < 0.001 |
| 8 | 0.659 | < 0.001 | 0.684 | < 0.001 | 0.670 | < 0.001 |

S1amp, amplitude of first heart sound; SBP, systolic blood pressure; dP/dt_max_, peak rate of increase in arterial pressure.

**Supplementary Table 5.** Correlations between s2 amplitude and hemodynamic variables by esmolol administration. Described for each case.

|  | **ΔS2amp and ΔSBP** | | **ΔS2amp and ΔPP** | | **ΔS2amp and ΔdP/dt_max_** | |
| --- | --- | --- | --- | --- | --- | --- |
| Case | R | *p*-value | r | *p*-value | r | *p*-value |
| 1 | 0.092 | 0.281 | 0.135 | 0.112 | 0.115 | 0.177 |
| 2^†^ | 0.813 | < 0.001 | 0.762 | < 0.001 | 0.768 | < 0.001 |
| 3 | 0.660 | < 0.001 | 0.640 | < 0.001 | 0.629 | < 0.001 |
| 4 | 0.657 | < 0.001 | 0.378 | 0.007 | 0.585 | < 0.001 |
| 5 | 0.888 | < 0.001 | 0.881 | < 0.001 | 0.819 | < 0.001 |
| 6 | 0.286 | 0.302 | -0.148 | 0.599 | -0.298 | 0.281 |
| 7 | 0.961 | < 0.001 | 0.960 | < 0.001 | 0.948 | < 0.001 |
| 8 | 0.866 | < 0.001 | 0.734 | < 0.001 | 0.838 | < 0.001 |

S1amp, amplitude of first heart sound; SBP, systolic blood pressure; dP/dt_max_, peak rate of increase in arterial pressure.

**Supplementary Table 6.** Correlations between s2 amplitude and hemodynamic variables induced by inferior-vena-cava clamping. Described for each case.

|  | **ΔS2amp and ΔSBP** | | **ΔS2amp and ΔPP** | | **ΔS2amp and ΔdP/dt_max_** | |
| --- | --- | --- | --- | --- | --- | --- |
| Case | r | *p*-value | r | *p*-value | r | *p*-value |
| 1 | 0.318 | < 0.001 | 0.280 | < 0.001 | 0.312 | < 0.001 |
| 2 | 0.596 | < 0.001 | 0.410 | < 0.001 | 0.149 | 0.002 |
| 3 | 0.656 | < 0.001 | 0.628 | < 0.001 | 0.586 | < 0.001 |
| 4 | -0.082 | 0.428 | -0.277 | 0.007 | -0.342 | < 0.001 |
| 5 | 0.406 | 0.002 | 0.475 | < 0.001 | 0.540 | < 0.001 |
| 6 | 0.924 | < 0.001 | 0.885 | < 0.001 | 0.846 | < 0.001 |
| 7 | 0.104 | 0.479 | 0.227 | 0.116 | 0.214 | 0.140 |
| 8 | 0.937 | < 0.001 | 0.862 | < 0.001 | 0.909 | < 0.001 |

S1amp, amplitude of first heart sound; SBP, systolic blood pressure; dP/dt_max_, peak rate of increase in arterial pressure.
